# Epidemiologic Characteristics and Clinical Significance of Respiratory Viral Infections Among Adult Patients Admitted to the Intensive Care Unit

**DOI:** 10.3389/fmed.2022.829624

**Published:** 2022-05-24

**Authors:** Jeong Yeon Kim, Kyung Sook Yang, Youseung Chung, Ki-Byung Lee, Jin Woong Suh, Sun Bean Kim, Jang Wook Sohn, Young Kyung Yoon

**Affiliations:** ^1^Division of Infectious Diseases, Department of Internal Medicine, Korea University College of Medicine, Seoul, South Korea; ^2^Department of Biostatistics, Korea University College of Medicine, Seoul, South Korea

**Keywords:** respiratory viral infections, viral diseases, intensive care units, pneumonia, epidemiology

## Abstract

**Background:**

The diagnosis of respiratory viral infections (RVIs) in critically ill patients is important for determining treatment options and adhering to infection-control protocols. However, data on the incidence and occurrence patterns of RVIs are scarce. We investigated the epidemiology and clinical impact of RVIs in critically ill patients.

**Methods:**

This retrospective observational study was conducted in a tertiary hospital in South Korea between November 2014 and September 2020. Adult patients (≥ 18 years of age) who tested positive for an RVI by multiplex polymerase chain reaction (mPCR) and were admitted to the intensive care unit (ICU) were included in the study. Clinical characteristics and outcomes were obtained by reviewing electronic medical records. Pearson's χ^2^ test and Fisher's exact test, Mann-Whitney U test was used to compare between groups of patients. Trend analysis and the χ^2^-based Q test was used to analyze test behavior of physicians performing mPCR test.

**Results:**

Among 22,517 patients admitted to the ICU during the study period, 2,222 (9.9%) underwent mPCR testing for an RVI. The median timing of mPCR testing after ICU admission was 1 day (IQR, 0–2). A total of 335 (15.1%) non-duplicative RVI-positive cases were included in the analysis. The incidence rate of RVIs in ICU patients was 30.45 per 10,000 patient-days. The most frequently detected RVI was influenza A (27.8%), followed by rhinovirus (25.4%). Thirty-two (9.6%) RVI-positive patients were diagnosed with upper respiratory infections, 193 (64.1%) with community-acquired, and 108 (35.9%) with hospital-acquired pneumonia. All-cause mortality and mortality related to respiratory tract infection (RTI) were 30.7% and 22.1%, respectively. The initial presentation of septic shock, requirement for mechanical ventilation, and lymphocytopenia were significant predictors of RTI-related mortality. Of the RVI-positive patients, 151 (45.1%) had nonviral coinfections and presented with higher clinical severity and longer hospital stays than patients infected solely with viral pathogens.

**Conclusion:**

The incidence of RVIs in ICU patients is common. ICU patients with RVIs had high mortality and frequently presented with coinfections with nonviral pathogens, which were associated with a higher clinical severity than sole RVI. Increased testing for RVIs will enhance infection-control efforts and improve patient care.

## Introduction

Research on the prevalence and prevention of respiratory viral infections (RVIs) is increasing. This is not only because of emerging infectious diseases such as the coronavirus disease 2019 and avian influenza but because of the recent advances and widespread use of multiplex molecular assays technology for diagnosing RVIs (1–3). Intensive care units (ICUs) have seen an increase in the prevalence of RVIs. Recent studies have reported that the prevalence of RVIs was as high as 16% −41% in critically ill ICU patients with community-acquired pneumonia (CAP) (4–6) and 17–29% in patients admitted with hospital-acquired pneumonia (HAP) (7–13).

The transmission of RVIs may be facilitated within hospital environments leading to nosocomial outbreaks (14–16). RVIs can be important determinants of adverse outcomes, especially among critically ill patients. These adverse outcomes are due to the direct viral immune evasion and the dysregulated immune response in these patients. RVIs can significantly contribute to morbidity, mortality, and healthcare costs (17). Identifying the dynamic epidemiologic characteristics of RVIs along with early RVI diagnoses have crucial implications for infection-control measures in ICU patients (18).

The clinical manifestations of RVIs range from asymptomatic or mild infections of the upper respiratory tract to severe pneumonia with respiratory failure. Clinicians challenge difficulties interpreting the clinical relevance of RVIs. They must distinguish between colonization, shedding, and true infection. Although largely disregarded in the past, viral detection is associated with higher ICU mortality, particularly for patients with influenza, parainfluenza, or the respiratory syncytial virus (RSV) (19–21). In addition, bacterial and fungal coinfections are present in 10–68 % ICU patients with RVIs (22, 23). Therefore, testing for RVIs both during and outside the influenza season as well as enhancing the understanding of the role of RVIs in critically ill patients can be useful for appropriate case management and containment of the nosocomial spread of RVIs.

Data on the epidemiologic characteristics and clinical impact of viruses other than the influenza virus and RSV in ICU patients are limited. A recent study found that the rhinovirus and human metapneumovirus are possible causative pathogens of severe pneumonia despite of their past view of weak pathogenicity (24). Additionally, a previous study suggested that RVIs play a significant role in immunocompetent patients (25). Thus, the objectives of this study were to 1) investigate the epidemiologic characteristics encompassing the overall incidence of RVIs, 2) assess the seasonal changes in RVIs, and 3) evaluate the clinical implications of RVIs during routine care over a period of six consecutive years.

## Materials and Methods

### Study Population

Our retrospective cohort study was conducted at a 1,048-bed tertiary care hospital in Seoul, Republic of Korea. This study was conducted from November 2014 to September 2020. All subjects were consecutive adult patients (≥ 18 years) who were admitted to the ICU and underwent multiplex polymerase chain reaction (mPCR) testing for RVIs. A respiratory virus identified by mPCR is always considered a pathogen of the respiratory tract, regardless of the type of specimen. If the same respiratory virus was recovered from a patient, only the first episode was included in our study to rule out long-term viral shedding. All patients were treated with established medical protocols. No additional mPCR tests were performed for this study.

The study protocol was approved before the study was initiated by the Institutional Review Board of Korea University Anam Hospital [No. 2021AN0445]. As this observational study did not deviate from routine medical practice, the requirement for informed consent was waived.

### Study Objectives

The primary endpoint was to investigate the prevalence of RVIs in ICUs. Secondary endpoints were to 1) describe the clinical practice of diagnostic testing for RVIs in patients admitted to ICUs, 2) determine the epidemiology of coinfections with viral and non-viral pathogens, 3) identify the associated risk factors, and 4) document the clinical outcomes.

### Study Definitions and Clinical Data

Each patient with positive mPCR results for an RVI was classified as either asymptomatic, as having an upper respiratory infection, CAP, or HAP. CAP and HAP were defined according to the American Thoracic Society/Infectious Disease Society of America guidelines (26). Severe pneumonia was diagnosed if invasive mechanical ventilation was required or septic shock occurred due to pneumonia (27). Septic shock was defined according to the third International Consensus Definitions for Sepsis and Septic Shock (Sepsis-3) (28). Patients were considered to have coinfections of the respiratory tract if sputum cultures, urinary antigen tests, or sputum PCR conducted within 48 h of mPCR for RVIs was positive.

The clinical data of each patient who underwent mPCR testing for RVIs were retrieved from a computerized hospital database. Clinical parameters for analysis included demographic and clinical characteristics (29–31), comorbid medical conditions (32), microbiological data, and treatment outcomes.

### Microbiological Evaluation

Patient specimens tested for RVIs varied and included nasopharyngeal swabs, endotracheal aspirates, and bronchoalveolar lavage (BAL) fluid according to the discretion of the attending physician. Respiratory viruses were detected by multiplex reverse-transcription PCR using an AdvanSure™ RV real-time PCR kit (AdvanSure; LG Life Sciences, Korea) from November 2014 to October 2017. An Anyplex^TM^ II RV16 Detection kit (Seegene Inc., Seoul, Korea) was used from November 2017 to September 2020. These kits simultaneously detect influenza A and B, human adenovirus, parainfluenza virus (types 1, 2, and 3), RSV (A and B), rhinovirus, human metapneumovirus, common human coronavirus (229E, OC43, NL63), and bocavirus.

Sputum culture isolates were analyzed by matrix-assisted laser desorption/ionization time-of-flight mass spectrometry (Bruker Diatonic GmbH, Bremen, Germany). Urinary antigen tests for *Streptococcus pneumoniae* and *Legionella pneumophila* serotype 1 were performed. Grocott's methenamine silver stain and *Pneumocystis jirovecii* PCR testing of respiratory samples were used to identify fungal coinfections when suspected.

### Statistical Analyses

The proportion of RVIs was calculated for each respiratory virus during the study period. The monthly incidence of each RVI was calculated as the number of cases per 10,000 patient-days.

Several groups of patients were compared. Patients who had pneumonia and those who did not, patients with CAP and those with HAP, patients who had coinfections with non-viral pathogens and those who had no coinfections were included in the evaluation. Categorical variables were compared using the Pearson's χ^2^ test and Fisher's exact test. The Kruskal–Wallis test confirmed that all continuous variables were distributed non-normally. Therefore, continuous variables were compared using the Mann–Whitney U test. Statistical significance was set at *p* < 0.05. To identify risk factors associated with mortality due to respiratory tract infection, multivariate logistic regression analysis using the backward stepwise variable selection based on likelihood ratio statistic was used. Hosmer–Lemeshow goodness-of-fit-test were performed to evaluate the models. Trend analysis of the rates of mPCR testing among patients admitted to the ICU was conducted using the Cochran–Armitage test. The χ^2^-based Q test was used to assess the heterogeneity of the rate of positive RVI results between years. Statistical analyses were performed using the IBM SPSS statistics program (version 23.0; IBM Corporation, Armonk, NY, USA).

## Results

### Diagnostic Testing for Respiratory Viral Infections

During the study period, 22,517 patients were admitted to the ICU, of which 2,222 (9.9%) underwent mPCR testing for RVIs. Of the patients admitted to the ICU, the proportion of subjects who underwent RVI testing by year was 2.0% (12/610) in 2014, 7.3% (255/3,506) in 2015, 8.9% (322/3,607) in 2016, 9.5% (372/3,910) in 2017, 11.5% (448/3,911) in 2018, 9.9% (414/4,198) in 2019, and 14.3% (399/2,784) in 2020 (Table 1). The proportion of patients who were admitted to the ICU that underwent mPCR testing showed an increasing trend year by year. (Cochran–Armitage test Z=-10.356, *P* < 0.001) (Table 1).

**Table 1:** Rates of respiratory virus diagnostic testing and incidence of respiratory virus infections.

**Variables**	**ICU-admission**	**mPCR test for RVIs**	**Results of mPCR test**					**Incidence of RVIs per 10,000 patient-days**
			**Positive**	**Negative**	**df**	**χ^2^**	***P*-value^**†**^**	
**Total**	22,517	2,222 (9.9)	335 (15.1)	1,887 (84.9)	6	27.769	**0.000104**	30.45
**Year**, ***n*** **(%)**								
2014*	601 (2.7)	12 (2.0)	2 (16.7)	10 (83.3)	1	0.026	0.872	6.23
2015	3,506 (15.6)	255 (7.3)	32 (12.5)	223 (87.5)	1	1.201	0.273	17.54
2016	3,607 (16.0)	322 (8.9)	49 (15.2)	273 (84.8)	1	0.012	0.913	26.30
2017	3,910 (17.4)	372 (9.5)	68 (18.3)	304 (81.7)	1	3.138	0.076	35.73
2018	3,911 (17.4)	448 (11.5)	77 (17.2)	371 (82.8)	1	1.681	0.195	40.19
2019	4,198 (18.6)	414 (9.9)	77 (18.6)	337 (81.4)	1	4.206	**0.04**	41.63
2020^§^	2,784 (12.4)	399 (14.3)	30 (7.5)	369 (92.5)	1	17.515	**<** **0.001**	22.65

*Df, difference for the Cochran–Armitage test; ICU, intensive care units; mPCR, multiplex polymerase chain reaction test; RVI, respiratory virus infection*.

*November 2014 to December 2014.

*^§^January 2020 to September 2020.*

*P-value for the heterogeneity test between positive and negative groups in the mPCR tests.*

*The bold values indicate P < 0.05*.

**Figure 1:**
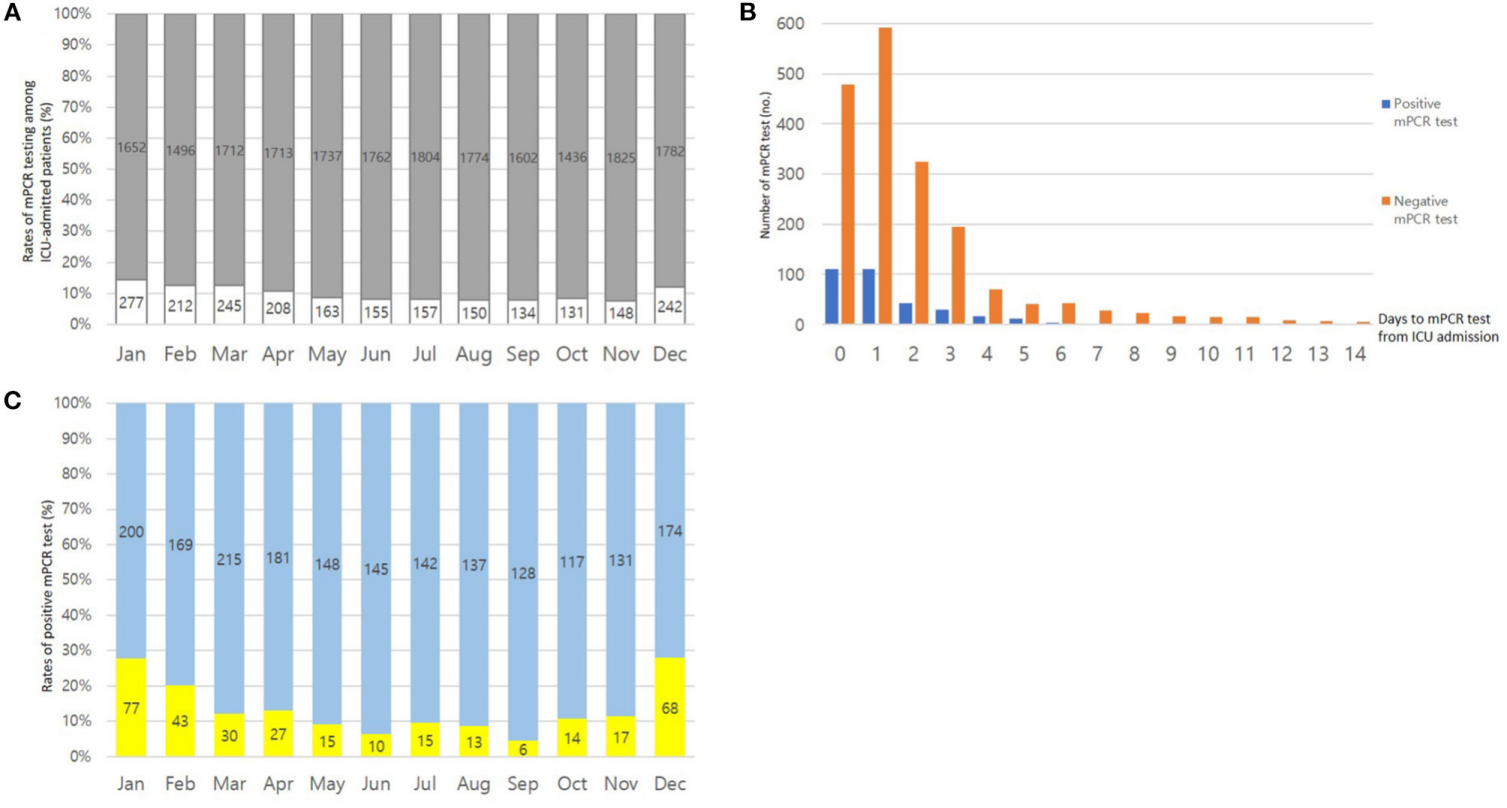
**(A)** Ratios of patients admitted to the intensive care unit (ICU): patients who underwent testing (white bar) and those who did not (gray bar). The total number of patients admitted to the ICU are shown above each column. **(B)** Timing of diagnostic testing for respiratory virus infections. **(C)** Seasonal proportions of respiratory virus test results that were positive (yellow bar) and negative (blue bar).

During the influenza season between November and April, 11.6% (1,332/11,512) of patients admitted to the ICU were tested for RVIs. Outside of the influenza season, 8.1% (890/11,005) of patients admitted to the ICU were tested for RVIs (*P* < 0.001) (Figure 1A). The median timing of virus diagnostic tests performed by the attending physician was 1 d (IQR, 0–2) after ICU admission. As shown in Figure 1B, more than half of the mPCR tests for RVIs were conducted within the first 48 h following ICU admission.

Of the patients who underwent mPCR testing for RVIs, the rate of positive test results was 16.7% in 2014, 12.5% in 2015, 15.2% in 2016, 18.3% in 2017, 17.2% in 2018, 18.6% in 2019, and 7.5% in 2020, respectively (Table 1). When the statistical heterogeneity test was performed, there was a significant difference in the rate of positive test results by year, particularly between 2019 and 2020 (χ^2^ = 27.769, *P* < 0.001). The monthly rate of positive mPCR tests for RVIs is presented in Figure 1C.

### Patient Characteristics

Of the 2,222 patients who underwent mPCR testing for RVIs, 335 (15.1%) positive cases were included in the analysis. Among these positive cases, RVIs were diagnosed, on average, 1.6 days after ICU admission. Patients who tested positive for RVIs had a median age of 74 (IQR, 63–82) years, and 182 (54.3%) were male. The median Acute Physiology and Chronic Health Evaluation II (APACHE-II) score and sepsis-related organ-failure assessment (SOFA) score on ICU admission was 26 (IQR, 19–33) and 5 (IQR, 2–7), respectively. The most common comorbidity was diabetes mellitus (29.3%), followed by congestive heart failure (24.2%) and chronic lung disease (20.3%). At least 64 (19.1%) patients were considered immunocompromised and experienced at least one of the following conditions: malignancy, bone-marrow transplantations, solid organ transplantation, or chemotherapy within the previous month. Other basal clinical characteristics are listed in Table 2.

**Table 2:** Demographic and clinical characteristics of patients with respiratory virus infections in intensive care units.

	**Total *n* = 335**	**Cases without pneumonia *n* = 34 (10.1%)**	**Cases with pneumonia**				*P*-value*
			**Total *n* = 301 (89.9%)**	**CAP *n* = 193 (64.1%)**	**HAP *n* = 108 (35.9%)**	***P*-value**	
Age (years), median (IQR)	74 [63–82]	74.5 [57.5–82.75]	74 [64–82]	74 [65–83]	73 [63.25–81]	0.537	0.952
Male, *n* (%)	182 (54.3)	10 (29.4)	172 (57.1)	103 (53.4)	69 (63.9)	0.077	0.003
**Distribution of respiratory viruses**							
Influenza A virus	93 (27.8)	10 (29.4)	83 (27.6)	63 (32.6)	20 (18.5)	**0.010**	0.841
Influenza B virus	13 (3.9)	1 (2.9)	12 (4)	10 (5.2)	2 (1.9)	0.223	1.000
Rhinovirus A/B/C	85 (25.4)	7 (20.6)	78 (25.9)	48 (24.9)	30 (27.8)	0.586	0.543
Common human coronavirus	52 (15.5)	9 (26.5)	43 (14.3)	22 (11.4)	21 (19.4)	0.061	0.063
Respiratory syncytial virus	44 (13.1)	1 (2.9)	43 (14.3)	26 (13.5)	17 (15.7)	0.609	0.064
Metapneumovirus	24 (7.2)	3 (8.8)	2 (0.7)	15 (7.8)	6 (5.6)	0.495	0.723
Parainfluenza virus	23 (6.9)	3 (8.8)	20 (6.6)	10 (5.2)	10 (9.3)	0.227	0.717
Human adenovirus	17 (5.1)	2 (5.9)	15 (5)	6 (3.1)	9 (8.3)	0.056	0.686
Bocavirus	3 (0.9)	1 (2.9)	2 (0.7)	0 (0)	2 (1.9)	0.128	0.275
Enterovirus	2 (0.6)	1 (2.9)	1 (0.3)	0 (0)	1 (0.9)	0.359	0.193
**Coinfection**, ***n*** **(%)**							
Bacterial	151 (45.1)	6 (17.6)	145 (48.2)	82 (42.5)	63 (58.3)	**0.008**	**0.001**
Fungal	4 (1.2)	0 (0)	4 (1.3)	1 (0.5)	3 (2.8)	0.134	1.000
**Specimen**, ***n*** **(%)**							**0.002**
Nasopharyngeal swab	232 (69.3)	32 (94.1)	200 (66.4)	145 (75.1)	55 (50.9)	**<** **0.001**	
Tracheal aspirate	67 (20)	2 (5.9)	65 (21.6)	33 (17.1)	32 (29.6)	**0.011**	
Bronchoalveolar lavage	36 (10.7)	0 (0)	36 (12)	15 (7.8)	21 (19.4)	**0.003**	
**Clinical severity on ICU admission, (mean** **±SD)**							
Septic shock, *n* (%)	174 (51.9)	9 (26.5)	165 (54.8)	104 (53.9)	61 (56.5)	0.718	**0.002**
APACHE-II scores	26 [19–33]	19 [14.5–29]	27 [19–34]	25 [18–34]	28 [23–34]	**0.034**	**0.001**
SOFA scores	5 [2–7]	49.5 [39.75–62.5]	65 [50–82]	5 [3–7]	5 [2–8]	0.871	**0.001**
SAPS 3 scores	63 [48–80]	3 [1.75–7]	5 [2–7]	64 [49–80]	67 [52–83]	0.431	**0.038**
**Treatment during ICU stay**, ***n*** **(%)**							**<** **0.001**
Nasal cannula oxygen	83 (24.8)	23 (67.6)	626 (20.8)	45 (23.3)	15 (13.9)	0.052	
Non-invasive ventilation	70 (20.9)	4 (11.8)	66 (21.9)	48 (24.9)	18 (16.7)	0.111	
Mechanical ventilation	174 (51.9)	7 (20.6)	167 (55.5)	93 (48.2)	74 (68.5)	**0.001**	
Extracorporeal membrane oxygenation	8 (2.4)	0 (0)	8 (2.7)	7 (3.6)	1 (0.9)	0.268	
Hemodialysis	27 (8.1)	4 (11.8)	66 (22.1)	17 (32.1)	10 (27.8)	0.815	0.275
**Underlying conditions**, ***n*** **(%)**							
Charlson comorbidity score, median [IQR]	5 [4–6]	5 [2.75–5.25]	5 [4–6]	5 [4–6]	5 [4–6]	0.747	0.320
Congestive heart failure	81 (24.2)	15 (44.1)	66 (21.9)	45 (23.3)	21 (19.4)	0.436	**0.006**
Chronic lung diseases	68 (20.3)	4 (11.8)	64 (21.3)	43 (22.3)	21 (19.4)	0.564	0.261
Diabetes mellitus	98 (29.3)	4 (11.8)	94 (31.2)	61 (31.6)	33 (30.6)	0.850	**0.027**
End-stage renal diseases	18 (5.4)	0 (0)	18 (6)	8 (4.1)	10 (9.3)	0.081	0.235
Liver cirrhosis	10 (3)	1 (2.9)	9 (3)	6 (3.1)	3 (2.8)	1.000	1.000
Cerebrovascular disease	47 (14)	5 (14.7)	42 (14)	25 (13)	17 (15.7)	0.503	0.800
Solid cancer	44 (13.1)	1 (2.9)	43 (14.3)	27 (14)	16 (14.8)	0.844	0.064
Hematologic malignancy	13 (3.9)	0 (0)	13 (4.3)	6 (3.1)	7 (6.5)	0.236	0.377
Solid organ transplant	4 (1.2)	0 (0)	17 (5.6)	0 (0)	4 (3.7)	**0.016**	1.000
Recent admission	97 (29)	9 (26.5)	88 (29.2)	33 (17.1)	55 (50.9)	**<** **0.001**	0.843
Receipt of chemotherapy	17 (5.1)	0 (0)	17 (5.6)	10 (5.2)	7 (6.5)	0.639	0.236
Recent surgery	8 (2.4)	0 (0)	8 (2.7)	2 (1)	6 (5.6)	**0.027**	1.000
**Laboratory findings, median (IQR)**							
White blood cells, /μL	10,500 [7,060–14,900]	9,450 [7,165–13,627.5]	10,620 [7,000–14,950]	10,600 [7,200–14,350]	10,710 [7,000–15,800]	0.490	0.551
Neutrophil cell count, /μL	8148 [5303–12165]	7323.5 [5302.75–9858.75]	8337.5 [5283.75–12,235.75]	7870 [5230–11,710]	8970 [5,400–13,690]	0.232	0.302
Neutropenia, *n* (%)	14 (4.2)	0 (0)	14 (4.7)	8 (4.1)	6 (5.6)	0.777	0.376
Lymphocyte count, /μL	834 [415–1374.75]	1120.5 [822.75–2051.75]	807.5 [400.5–1308.75]	820 [425–1485]	760 [280–1600]	**0.036**	**0.003**
Lymphocytopenia, *n* (%)	197 (58.8)	13 (38.2)	184 (61.1)	111 (57.5)	73 (68.2)	0.083	**0.011**
Platelets, × 10^3^/μL	176 [117–247]	192 [135.75–221.25]	175 [114.5–247]	177 [123–246]	165.50 [87.50–252]	0.259	0.195
Creatinine, mg/dL	1.08 [0.79–1.59]	1.18 [0.8125–1.7075]	1.07 [0.775–1.59]	1.10 [0.8–1.55]	1.01 [0.69–1.82]	0.367	0.719
C-reactive protein, mg/L	102.49[34.22–191.75]	20.175 [5.185–102.605]	118.14 [43.94–199.185]	100.02 [29.61–191.68]	133.94 [67.94–223.59]	**0.008**	**<** **0.001**
C-reactive protein ≥ 100 mg/L, *n* (%)	173 (51.6)	8 (23.5)	165 (54.8)	97 (50.3)	68 (63.0)	**0.040**	**<** **0.001**
Procalcitonin, ng/mL	0.61 [0.20–2.97]	0.2850 [0.0830–1.67]	0.6895 [0.2155–3.1775]	0.54 [0.18–2.97]	1.17 [0.36–5.11]	**0.040**	**0.039**
Procalcitonin ≥ 0.5 ng/mL	162 (48.4)	9 (26.5)	153 (50.8)	74 (41.1)	49 (53.3)	0.071	**0.026**
**Outcomes**							
ICU days, median (IQR)	5 [3–9]	3.5 [2–6]	5 [3–9]	4 [3–8]	7.5 [4–12]	**<** **0.001**	**0.003**
Total hospital days, median (IQR)	21 [12–36]	21 [12.5–37]	15.5 [9–33]	17 [10–28]	31.5 [16–52.5]	**<** **0.001**	0.069
Hospital days after RVIs diagnosis, median (IQR)	16 [9–29]	13 [7.75–24.5]	17 [9–29.5]	15 [9–26]	18 [10–37.75]	**0.087**	0.243
28-day mortality, *n* (%)	64 (19.1)	2 (5.9)	62 (20.6)	39 (20.2)	23 (21.3)	0.882	**0.062**
All-cause mortality, *n* (%)	103 (30.7)	4 (11.8)	99 (32.9)	52 (26.9)	47 (43.5)	**0.003**	**0.017**
RTI-related mortality, *n* (%)	74 (22.1)	0 (0)	74 (24.6)	39 (20.2)	35 (32.4)	**0.025**	**0.001**

*CAP, community-acquired pneumonia; HAP, hospital-acquired pneumonia; IQR, interquartile range; ICU, intensive care unit; APACHE-II, Acute Physiology and Chronic Health Evaluation II; SOFA, sepsis-related organ failure assessment; RTI, respiratory tract infections; SAPS 3, Simplified Acute Physiology Score 3. P-value for the heterogeneity test between positive and negative groups in the mPCR tests*.

*P-value for the Pearson's χ^2^ test, Fisher's exact test, or Mann–Whitney U test between cases without pneumonia and pneumonia cases.

*The bold values indicate P < 0.05*.

### Clinical Characteristics

A total of 335 non-duplicate mPCR-positive cases were identified. The clinical manifestations of these cases were categorized as asymptomatic infection (*n* = 2, 0.6%), upper respiratory infection (*n* = 32, 9.6%), pneumonia [*n* = 301, 89.9%; CAP (57.6%, 193/335), and HAP (32.2%, 108/335]). Among 108 HAP patients, 69 (63.9%) patients either developed RVI symptom 14 days after hospital admission or were transferred from other hospital. The median length of stay in the ICU or hospital after diagnosis with an RVI was 5 (3–9) days and 21 (12–36) days, respectively. Of the patients who tested positive for RVIs, 174 (51.9%) required mechanical ventilation. The 28-day mortality and all-cause in-hospital mortality rates were 19.1% and 30.7%, respectively.

Among the mPCR-positive cases, comorbidity with diabetes mellitus was more common in patients with pneumonia than in patients without pneumonia (Table 2). There were significant differences in the clinical severity evaluated using the APACHE-II, SOFA, and Simplified Acute Physiology Score 3 (SAPS 3). The clinical outcomes were evaluated by comparing mortality in patients with pneumonia and those without pneumonia (Table 2).

The results of the comparison of the demographic and clinical characteristics of patients with CAP and those with HAP are shown in Table 2. Of the 108 patients diagnosed with HAP, 39 (36.1%) were transferred to our ICU from other hospitals. Patients with HAP had a greater association with bacterial coinfection [63/108 (58.3%) vs. 82/193 (42.5%), *P* = 0.008] and had higher all-cause in-hospital mortality rates [47/108 (43.5%) vs. 52/108 (26.9%), *P* = 0.003] than patients with CAP.

### Prevalence and Distribution of Respiratory Viral Infections

**Figure 2:**
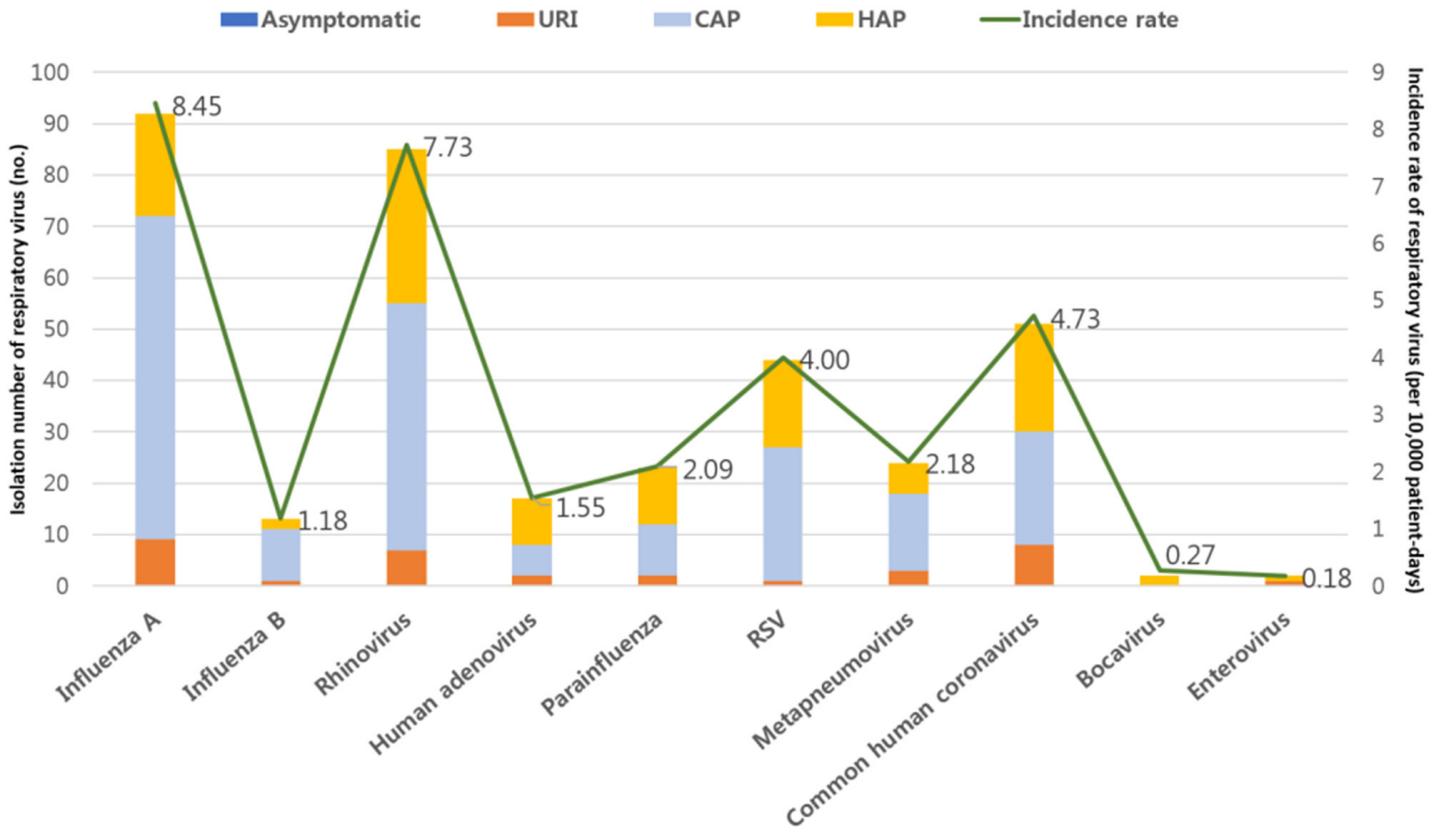
Prevalence rates of respiratory virus infections (RVIs) in patients admitted to the intensive care unit. URI, upper respiratory tract infections; CAP, community-acquired pneumonia; HAP, hospital-acquired pneumonia.

The specimens collected for detecting respiratory viral infections were distributed as follows: nasopharyngeal swabs (*n* = 232, 69.3%), endotracheal aspirates (*n* = 67, 20.0%), and BAL fluid (*n* = 36, 10.7%). A total of 356 respiratory viruses were identified in 335 patients. Twenty-three patients (6.9%) were infected with two or more types of respiratory virus. The overall prevalence rate of RVIs in the ICU was 30.45 per 10,000 patient-days. Influenza A virus (*n* = 93) was the most commonly detected virus with a prevalence rate of 8.45 per 10,000 patient-days, followed by rhinovirus (*n* = 85, 7.73 per 10,000 patient-days), common human coronavirus (*n* = 52, 4.73 per 10,000 patient-days), RSV (*n* = 44, 4.00 per 10,000 patient-days), human metapneumovirus (*n* = 24, 2.18 per 10,000 patient-days), parainfluenza (*n* = 23, 2.09 per 10,000 patient-days), human adenovirus (*n* = 17, 1.55 per 10,000 patient-days), influenza B (*n* = 13, 1.18 per 10,000 patient-days), bocavirus (*n* = 3, 0.27 per 10,000 patient-days), and enterovirus (*n* = 2, 0.18 per 10,000 patient-days) (Figure 2).

**Figure 3:**
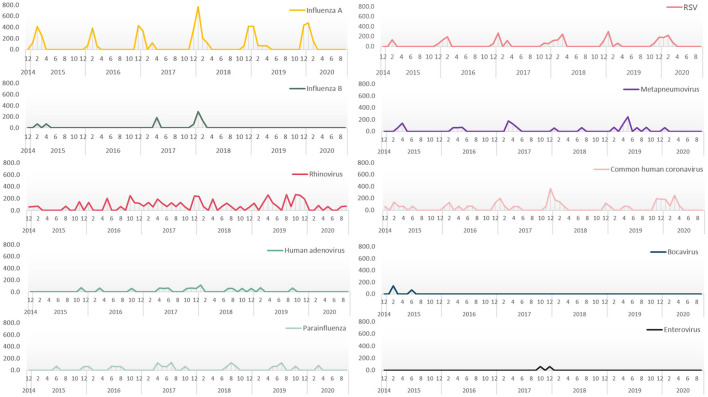
Monthly prevalence rates showing seasonality of respiratory virus infections in patients admitted to the intensive care unit.

**Figure 4:**
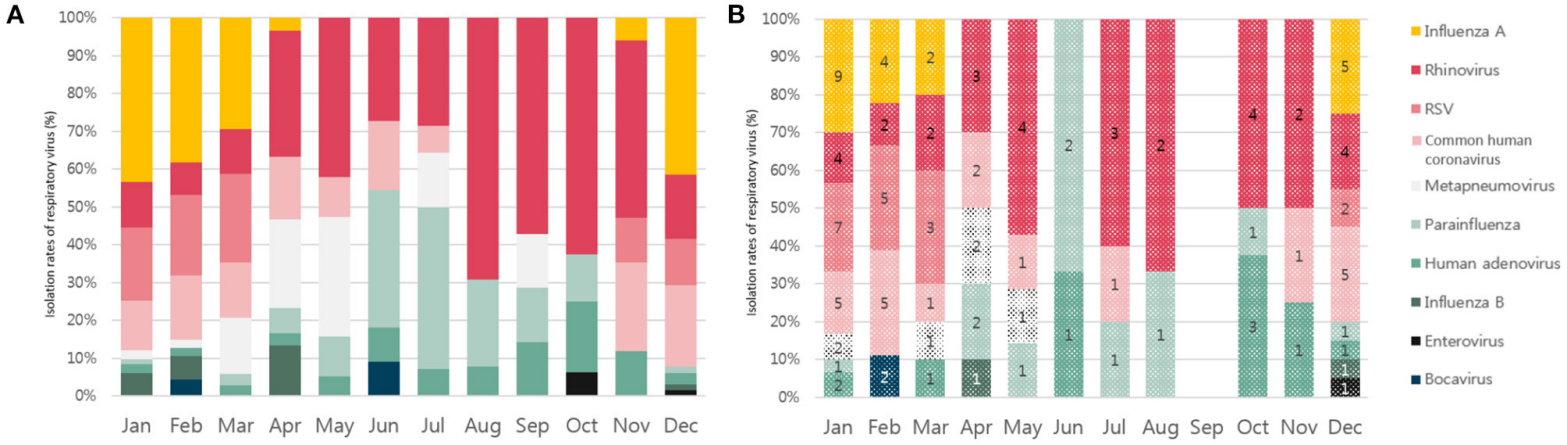
**(A)** Seasonal distribution of the detection of each respiratory virus in the intensive care unit (ICU) patients with respiratory virus infections. **(B)** Seasonal distribution of the detection of each respiratory virus in the ICU patients with hospital-acquired pneumonia.

Seasonality was noted for influenza A, with a prevalence rate that peaked from December to February every year (Figure 3). RSV showed a peak prevalence from November to April. In the case of parainfluenza infections, which were typically parainfluenza 3 (16/23, 69.6%), repetitive peaks occured from late spring to summer. Some small peaks occurred in winter. Conversely, RVIs caused by rhinovirus, human adenovirus, and human metapneumovirus occurred consistently throughout the year without clear seasonality. In ICU patients with RVIs, the distribution of monthly occurrences by the type of RVI is shown in Figure 4A. The distribution of monthly occurrences by the type of RVI in ICU patients with HAP was similar to that of the overall ICU population (Figure 4B).

The all-cause in-hospital mortality rate of each RVI were as follows: 32.2% for influenza A, 34.6% for rhinovirus, 29.7% for RSV, and 25.0% for common human coronavirus. There was no statistically significant difference according to the type of RVI. Unlike other respiratory viruses, influenza A was more frequently detected in patients with CAP than in those with HAP (Table 2).

### Coinfections of Respiratory Tract With Nonviral Pathogens

Nonviral coinfections of respiratory tract were identified in 151 patients (45.1%) (Table 3). Among them, 133 (88.1%) cases were identified with induced sputum and 18 (11.9%) cases were identified with BAL fluid. However, there were no statistic difference of proportion of BAL specimen between viral only group and nonviral coinfected group. The most frequently isolated bacterial pathogens were *Staphylococcus aureus* (*n* = 47, 25.5%), followed by *Streptococcus pneumoniae* (*n* = 40, 21.7%), and *Acinetobacter baumannii* (*n* = 23, 12.5%) (Table 3). Patients with nonviral coinfections had higher APACHE-II scores [28 (IQR, 21–36) vs. 24 (IQR, 17–31), *P* = 0.001] and SAPS 3 scores [67 (IQR 52–85) vs. 59.5 (IQR, 45–75.5), *P* = 0.002] on ICU admission, than patients with only viral infections (Table 4). Laboratory findings indicated that C-reactive protein ≥ 100.0 mg/L [81/184 (44.0%) vs. 93/151 (60.9%), *P* = 0.002] and procalcitonin (≥ 0.5 ng/mL) [79/162 (48.8%) vs. 83/136 (61.0%), *P* = 0.036] were more frequent in patients with nonviral coinfections than in patients with only viral infections (Table 4). Patients with nonviral coinfections had longer total hospital stays [23 days (IQR, 15–43) vs. 18 days (IQR, 10–32), *P* < 0.001] than patients with only viral infections (Table 4).

**Table 3:** Distribution of coinfections with non-viral pathogens and respiratory virus infections in intensive care units.

Identified organism*	**Influenza A virus (*n* = 93)**	**Influenza B virus (*n* = 13)**	**Rhinovirus A/B/C (*n* = 85)**	**Commonhuman coronavirus (*n* = 52)**	**Respiratory syncytial virus (*n* = 44)**	**Metapneumovirus (*n* = 24)**	**Parainfluenza virus (*n* = 23)**	**Human adenovirus (*n* = 17)**	**Bocavirus (*n* = 3)**	**Enterovirus (*n* = 2)**
*Streptococcuspneumoniae*^§^ (*n* = 40)	5	2	12	5	7	7	2	1		
*Streptococcus pyogenes* (*n* = 1)				1						
*Staphylococcus aureus* (*n* = 47)	19	5	5	5	8	2	3	3		1
*Haemophilus influenzae* (*n* = 6)	2		1	1		1	1			
*Moraxella catarrhalis* (*n* = 1)				1						
*Legionellapneumophila*^†^ (*n* = 1)	1									
*Achromobacter xylosoxidans* (*n* = 1)				1						
*Klebsiella pneumoniae* (*n* = 14)	1	1	6		5	1		2		
*Escherichia coli* (*n* = 15)	4	1	6	3	2		1	1		
*Enterobacter aerogenes* (*n* = 2)	1							1		
*Serratia marcescens* (*n* = 3)			3							
*Pseudomonas aeruginosa* (*n* = 18)	3	1	5	3	1	3	2	1		1
*Providencia stuartii* (*n* = 2)	1		1							
*Acinetobacter baumannii* (*n* = 23)	9	1	3	3	1		3	4		1
*Stenotrophomonas maltophilia* (*n* = 3)			1				1	1		
*Mycoplasmapneumoniae*^¶^ (*n* = 1)			1							
*Pneumocystis jirovecii* (*n* = 3)			2				1	1		
*Aspergillus species* (*n* = 1)					1					
*Mycobacterium tuberculosis* (*n* = 2)						1	1	1		
None	54	5	46	33	26	15	12	6	3	1

*More than one pathogen was detected in some patients.

*§ Thirteen cases were Streptococcus urinary Ag positive or trace.*

*Detected by Legionella urinary Ag.*

*^¶^Detected by sputum mycoplasma PCR*.

**Table 4:** Comparison between patients with only respiratory viral infections and patients with coinfections.

	**Total (*n* = 335)**	**Viral only (*n* = 184, 54.9%)**	**Coinfections (*n* = 151, 45.1%)**	***P*-value**
**Age (years), median (IQR)**	74 [63–82]	73.5 [62–82.75]	75 [64–81]	0.839
**Male**, ***n*** **(%)**	182 (54.3)	96 (52.2)	86 (57.0)	0.382
**Specimen**, ***n*** **(%)**				
Nasopharyngeal swab	232 (69.3)	137 (74.5)	95 (62.9)	**0.023**
Tracheal aspirate	67 (20.0)	29 (15.8)	38 (25.2)	**0.042**
Bronchoalveolar lavage	36 (10.7)	18 (9.8)	18 (11.9)	0.530
**Clinical severity on ICU admission, (mean** **±SD)**				
Septic shock, n (%)	174 (51.9)	85 (46.2)	89 (58.9)	**0.021**
APACHE-II scores	26 [19–33]	24 [17–31]	28 [21–36]	**0.001**
SOFA scores	5 [2–7]	4 [2–7]	5 [2–8]	0.119
SAPS 3 scores	63 [48–80]	59.5 [45–75.5]	67 [52–85]	**0.002**
**Treatment during ICU stay**, ***n*** **(%)**				
Nasal cannula oxygen	83 (24.8)	61 (33.2)	22 (14.6)	**<** **0.001**
Non-invasive ventilation	70 (20.9)	40 (21.7)	30 (19.9)	0.688
Mechanical ventilation	174 (51.9)	79 (42.9)	95 (62.9)	**<** **0.001**
Extracorporeal membrane oxygenation	8 (2.4)	4 (2.2)	4 (2.6)	1.000
Renal replacement therapy	27 (8.1)	17 (27.0)	12 (31.6)	0.655
**Underlying Conditions**, ***n*** **(%)**				
Charlson comorbidity score, median [IQR]	5 [4–6]	5 [3–6]	5 [4–7]	**0.001**
Congestive heart failure	81 (24.2)	43 (23.4)	38 (25.2)	0.702
Chronic lung diseases	68 (20.3)	31 (16.8)	37 (24.5)	0.083
Diabetes mellitus	98 (29.3)	52 (28.3)	46 (30.5)	0.659
End-stage renal diseases	18 (5.4)	8 (4.3)	10 (6.6)	0.358
Liver Cirrhosis	10 (3.0)	5 (2.7)	6 (4.0)	0.552
Cerebrovascular disease	47 (14.0)	29 (15.8)	18 (11.9)	0.314
Solid Cancer	44 (13.1)	24 (13.0)	20 (13.2)	0.957
Hematologic malignancy	13 (3.9)	9 (4.9)	4 (2.6)	0.290
Solid organ Transplant	4 (1.2)	1 (0.5)	3 (2.0)	0.331
Recent admission	97 (29.0)	56 (30.4)	41 (27.2)	0.510
Receipt of chemotherapy	17 (5.1)	9 (4.9)	8 (5.3)	0.866
Recent surgery	8 (2.4)	3 (1.6)	5 (3.3)	0.475
**Biochemistry, median [IQR]**				
White blood cells, /μL	10,500 [7,060–14,900]	10,500 [7,095–14,600]	10,340 [7,000–15,100]	0.764
Neutrophil cell count, /μL	8,148 [5303–12165]	8,030 [5312.5–11590]	8,280 [5190–12452.5]	0.396
Lymphocyte count, /μL	834 [415–1374.75]	945 [475–1607.5]	745 [400–1165]	**0.002**
Platelets, × 10^3^/μl	176 [117–247]	178 [121–247.75]	170 [108–243]	0.245
Creatinine, mg/Dl	1.08 [0.79–1.59]	1.10 [0.82–1.57]	1.04 [0.77–1.62]	0.715
C-reactive protein, mg/L	102.49 [34.22–191.75]	87.08 [23.87–169.63]	150.63 [46.63–235.21]	**<** **0.001**
C-reactive protein ≥ 100 mg/L, n (%)	173 (51.6)	81 (44.0)	93 (60.9)	**0.002**
Procalcitonin, ng/mL	0.61 [0.20–2.97]	0.43 [0.18–2.44]	0.96 [0.25–6.01]	**0.041**
Procalcitonin ≥ 0.5 ng/mL	162 (48.4)	79 (48.8)	83 (61.0)	**0.036**
**Outcomes**				
ICU days, median [IQR]	5 [3–9]	4 [3–8]	5 [3–10]	0.089
Total hospital days, median [IQR]	21 [12–36]	18 [10–32]	23 [15–43]	**<** **0.001**
Hospital days after RVIs diagnosis, median (IQR)	16 [9–29]	14 [8–26.75]	19 [10–33]	**0.011**
28-day mortality, *n* (%)	64 (19.1)	35 (19.0)	29 (19.2)	1.000
All-cause mortality, *n* (%)	103 (30.7)	54 (29.3)	49 (32.5)	0.540
RTI-related mortality, *n* (%)	74 (22.1)	39 (21.2)	35 (23.2)	0.693

*CAP, community-acquired pneumonia; HAP, hospital-acquired pneumonia; IQR, interquartile range; ICU, intensive care unit; APACHE-II, Acute Physiology and Chronic Health Evaluation II; SOFA, sepsis-related organ failure assessment; SAPS 3, Simplified Acute Physiology Score 3; RTI, respiratory tract infections. The bold values indicate P < 0.05*.

### Predictors Associated With Mortality Due to Respiratory Tract Infection

By the multivariate logistic regression analysis, the initial presentation of septic shock (odds ratio [OR] 4.59; 95% confidence interval [CI], 1.14–18.57), requirement for mechanical ventilation (OR 9.19; 95% CI, 2.06–40.88), and lymphocytopenia (OR 9.35; 95% CI, 1.61–54.36) were significantly associated with mortality related to respiratory tract infections in ICU patients with RVIs. *P*-values for the Hosmer–Lemeshow goodness-of-fit test were >0.05 (*P* = 0.652). Hence, there was no significant evidence of a lack of fit for any of the final models.

## Discussion

We investigated the epidemiology of RVIs in patients admitted to the ICU. Real-time mPCR tests for RVIs were performed in 9.9% of patients who were admitted to the ICU, and 15.1% of these patients tested positive for RVIs. The incidence of several RVIs identified during the study period showed seasonality. Among patients with positive RVI results, 89.9% had pneumonia. The 28-day mortality rate was as high as 19.1%. Furthermore, nearly half (45.1%) of the patients with RVIs were coinfected with non-viral pathogens. These were associated with increased clinical severity. In ICU patients with RVIs, the initial presentation of septic shock, requirement for mechanical ventilation, and lymphocytopenia were significant predictors of RTI-related mortality.

Our study demonstrated that 9.9% of ICU patients were tested for RVIs. The median timing of virus diagnostic testing was 1 d (IQR, 0–2) after ICU admission. More than half of the mPCR tests for RVIs were performed during the first 48 h following ICU admission (Figure 1B). A previous study also reported that virus tests were typically ordered on the day of ICU admission in cases suspected of RTIs (24). Although international guidelines are unclear regarding whether all critically ill patients with suspected pneumonia should be tested for RVIs, routine testing for RVIs should be actively considered in patients with severe pneumonia, especially during the influenza season. Importantly, our study showed an increasing trend in the number of tests for RVIs ordered during the winter season (Figure 1A).

Considering that testing for RVIs is mainly performed within the first 48 h of admission to the ICU, it can be inferred from the present study that the frequency of testing for RVIs in HAP patients is relatively low compared with that in CAP patients. However, in patients with RVIs, the frequency of RVIs by type showed no difference between CAP and HAP patients. This is similar to the results of previous studies, with the exception of influenza A (33). Presently, descriptions of nosocomial respiratory virus infections are scarce. Available studies report that rhinovirus and influenza virus are common hospital-acquired viruses. This is partially consistent with our findings (33). Previous studies have also reported that patients with HAP have a considerable risk of developing RVIs (8, 34).

The all-cause in-hospital mortality rate of each RVI was 32.2% for influenza A virus, 34.6% for rhinovirus, 29.7% for RSV, and 25.0% for common human coronavirus. A recent study suggested a causative role of rhinovirus for the development of severe pneumonia over other RVIs in immunocompetent patients (35). Additionally, influenza, parainfluenza, and RSV have been previously identified as prevalent pathogens in ICU non-survivors (6). Testing for non-influenza viruses in ICUs may be useful for determining the spread of infection and predicting patient outcomes. However, further studies are needed to determine whether these respiratory viruses originate from critically ill patients or are community-acquired from non-hospitalized individuals. Indeed, according to the guidelines for isolation precautions, some infectious-disease experts recommend a droplet isolation protocol for patients with RVIs to mitigate the spread of RVIs in hospital settings (36). Finally, a positive mPCR test in an ICU patient does not necessarily indicate critical virus-associated illness. There are no specific antiviral agents for infections caused by non-influenza viruses. Therefore, future studies should explore how early identification of non-influenza RVIs can be used to optimize patient outcomes.

In this study, coinfections with non-viral pathogens were observed in 45.1% of ICU patients with RVIs. Coinfections were associated with significantly higher morbidity than morbidity in patients with viral infection alone (Table 4). Previously reported rates of viral–bacterial coinfections range from 10 to 68% in hospitals and ICUs (20). Considering this epidemiologic challenge, selective testing for RVIs in ICU patients may result in the under-diagnosis of RVIs, particularly in those with pneumonia. Thus, respiratory viral testing of ICU patients with a suspected RVI is warranted independent of symptom severity. In agreement with findings from previous studies, our findings also showed that bacterial coinfections could be distinguished by assessing the serum biomarker C-reactive protein or procalcitonin (37, 38). In the present study, ICU patients with RVIs and coinfections of non-viral pathogens showed a significantly higher clinical severity (Table 4). This is in agreement with a previous study that suggested that viral–bacterial coinfections are an independent risk factor for ICU and in-hospital mortality (39). In our study, the most frequently isolated bacterial pathogen was *S. aureus* (*n* = 47, 25.5%), followed by *S. pneumoniae* (*n* = 40, 21.7%), and *A. baumannii* (*n* = 23, 12.5%). *S. pneumoniae* and *S. aureus* are the most commonly isolated bacterial pathogens in patients with CAP (40), while *S. aureus* and gram-negative bacilli are the predominant pathogens isolated from HAP patients (8).

This study has several limitations. First, this study included adult ICU patients with diverse accompanying diseases in a single institution, preventing any conclusions regarding other populations. Second, the diagnosis of RVIs was defined by the positive results of mPCR tests of various clinical samples ordered according to the discretion of the attending physician. Therefore, selection bias due to ICU patients who did not undergo mPCR testing for RVIs could not be excluded from this study. However, this study demonstrates the contemporary behavior of physicians diagnosing RVIs in the ICU and the clinical spectrum of RVI patients. Third, mPCR tests are unable to determine virus viability. Therefore, it is not clear whether certain RVIs are community-acquired infections or nosocomial infections. In particular, confirming that RVIs isolated from patients with HAP are nosocomial infections is difficult. Fourth, more than half of the specimens collected were nasopharyngeal swabs and may not have identified a potentially lower respiratory-tract infection. Therefore, determining whether the virus identified in these cases contributed to the development of pneumonia is difficult.

In conclusion, our study showed that the incidence of RVIs in ICU patients is common. ICU patients with RVIs had high mortality and revealed a high frequency of coinfection with bacterial pathogens. This was associated with a relatively high clinical severity. Thus, our findings provide additional evidence in support of more active testing for RVIs to improve patient outcomes and to enhance infection-control efforts.

## Data Availability Statement

The datasets used and analyzed during the current study are available from the corresponding author on reasonable request.

## Ethics Statement

The studies involving human participants were reviewed and approved by the Institutional Review Board of Korea University Anam Hospital [No. 2021AN0445]. Written informed consent for participation was not required for this study in accordance with the national legislation and the institutional requirements.

## Author Contributions

YY conceived the study. YY and JK designed and performed the study and wrote the manuscript. JK and KY analyzed the data. YC, K-BL, SK, and JS collected clinical data. All authors contributed to the article and approved the submitted version.

## Funding

This research was supported by grants from Korea University Anam Hospital, Seoul, Republic of Korea, and the Korea Health Technology R&D Project through the Korea Health Industry Development Institute (KHIDI), funded by the Ministry of Health and Welfare, Republic of Korea (grant number: HI20C0384). The funding source had no role in the study design, data collection and analysis, decision to publish, or preparation of the manuscript.

## Conflict of Interest

The authors declare that the research was conducted in the absence of any commercial or financial relationships that could be construed as a potential conflict of interest.

## Publisher's Note

All claims expressed in this article are solely those of the authors and do not necessarily represent those of their affiliated organizations, or those of the publisher, the editors and the reviewers. Any product that may be evaluated in this article, or claim that may be made by its manufacturer, is not guaranteed or endorsed by the publisher.
